# Virtual medical education accreditation survey visits and viability for the future

**DOI:** 10.12688/mep.19160.2

**Published:** 2023-01-20

**Authors:** Lori M. DeShetler

**Affiliations:** 1 Medical Education, The University of Toledo, Toledo, OH, 43614, USA

**Keywords:** Medical school accreditation, virtual survey visit, undergraduate medical education, COVID-19, Liaison Committee on Medical Education, LCME

## Abstract

Coronavirus disease 2019 (COVID-19) drastically disrupted daily life and abruptly forced curricular modifications in undergraduate medical education. Despite level of preparedness, medical schools moved instruction online for students to access remotely. Similarly, accreditation visits by the Liaison Committee for Medical Education (LCME) were also moved from in-person to virtual formats during these chaotic times. Little guidance was available to transition to the new process. Medical schools that were scheduled for a virtual survey visit were required to pivot without tried experience on how to prepare for and conduct these high stakes online visits. New processes needed to be developed to successfully navigate a virtual accreditation visit. To date, there has been nothing in the literature from those who have participated on LCME teams nor from medical schools who have undergone a virtual survey visit. This article accounts for one medical school’s experience from its 2021 LCME virtual visit and makes recommendations to consider when planning for such a significant event. The future of virtual visits is taken into account as this method has its benefits including elimination of travel and the associated time and cost. Yet, the perspectives from others who have participated in a virtual LCME accreditation visit should be studied. While the LCME will return to in-person visits in 2022-23, it is important for medical schools to share their experiences and lessons learned from their virtual accreditation visit should the need arise to reinstate virtual visits in the future.

## Introduction

The coronavirus disease 2019 (COVID-19) pandemic uprooted the daily sense of normalcy throughout society and displaced routines. Undergraduate medical education was forced to make modifications to curriculum. Didactic sessions were moved online for students to access remotely, although not all institutions were fully prepared for this transition to online learning ^1^. Examination methods were changed to eliminate in-person test administration, and some schools adopted open-book approaches ^2^. Anatomy lab experiences were moved to e-learning platforms, utilizing virtual meeting rooms for small and large-group sessions to deliver anatomy instruction ^3^. Student rotations at clinics and hospitals were halted as medical schools were directed by the Association of American Medical Colleges and the Liaison Committee on Medical Education (LCME) to suspend clinical activities for safety reasons ^4^. Once students were permitted to continue in clinical settings, many schools were faced with shortening the duration of rotations to compensate for the time lost during the stay-at-home order.

Medical schools had to quickly pivot during the pandemic to modify the curriculum and best support students during these challenging times. Schools that were planning for accreditation survey visits were also forced to switch gears due to COVID-19. In-person visits were no longer feasible, so online visits were instituted. An accreditation visit is an extremely important, high stakes event that requires a significant amount of planning. With the sudden shift to a virtual visit and the lack of known best practices, medical schools were left on their own to navigate the new online process. A few suggestions from peers surfaced in the Accreditation Prep & Quality Improvement Professional Learning Community (an online discussion group for educators involved with accreditation), but information was scant in these unprecedented times. One thing was certain: a different approach was needed to accomplish a virtual visit. With this new method, allocation of new or additional resources would be needed.

There are many articles in the nursing discipline about virtual accreditation visits. Anderson *et al.* ^5^ offered a bulleted list of key lessons learned in executing a successful virtual visit, which included partnering with information systems/technology to support each session, dress rehearsing the technology with information technology (IT) to assist with technological issues, testing for dead spots and inconsistent Wi-Fi, reminding staff to unmute, selecting a teleconferencing platform to optimize the experience, etc. The authors also discussed the importance of participants conveying the same level of energy in a virtual meeting as they would have provided in-person. Similarly, Phan and Radovich ^6^ suggested tips for a successful virtual site visit. The authors reported that a first step should be discussion between the director and lead appraiser (visitor) to agree upon the agenda, virtual platform, and expectations for the virtual visit.

From the field of pharmacy, Clarke *et al.* ^7^ explored the opportunity for quality assurance site visits to be conducted virtually, as an innovative method, prior to the need caused by the pandemic. They studied the difference in preceptor perceptions of virtual visits versus onsite visits and found that preceptors felt virtual visits were an acceptable option. In fact, some preceptors highly preferred this method. The authors also noted that interactions among those participating were similar across virtual and onsite methods. However, as Howe ^8^ points out, it is important to consider the limitations of a virtual scenario in which visitors cannot directly view the campus facilities, workflow, and other aspects beyond the view of the participant’s camera.

A review of relevant literature was conducted via PubMed to understand how medical schools coped with and learned from their virtual accreditation visit, or if there were any insights from those who participated as an LCME team member conducting an online survey visit. The search included the following keywords: medical school, undergraduate medical education, covid-19, LCME or Liaison Committee on Medical Education, medical school accreditation, and virtual accreditation visits. Despite the number of articles that surfaced from other disciplines ^5– 8^, at the time of writing this article there are yet to be any studies about the impact of COVID-19 on medical education accreditation visits by the LCME. This gap in the literature is important to recognize due to 1) the significance that an accreditation visit holds for medical schools, 2) the amount of preparation needed to transition from an onsite to a virtual visit, and 3) the potential for future pandemics or other disruptions that necessitate a virtual visit.

In medical education, accreditation visits historically have entailed in-person, multi-day sessions. Preparation for an accreditation survey visit by the LCME typically begins two years in advance. The LCME posts guidelines and procedures on its website to assist schools preparing for a visit ^9^. According to these documents, medical schools appoint a Faculty Accreditation Lead (FAL) and Survey Visit Coordinator (SVC) who plan the visit and organize committees to conduct a self-study and draft the survey package. In years prior to the pandemic, the LCME would send a team of peers to visit the medical school campus to evaluate the program. A considerable amount of expenses was incurred by the survey team for airfare and lodging, and the medical school was responsible for funding the team’s ground transportation and meals (breakfast and lunch) during the visit.

The University of Toledo College of Medicine and Life Sciences was in the midst of planning for its 2021 survey visit when the pandemic hit. COVID-19 was rapidly spreading and thus, face-to-face contact and travel was halted across the globe in spring 2020. To comply with the stay-at-home order, we moved all accreditation task force and subcommittee meetings from in-person to remote. The review and discussion of survey package documents all had to be conducted virtually. This meant committee members were required to spend long hours in front of a screen instead of meeting face-to-face in a board room. Despite the screen fatigue, an advantage was the ability for leaders to conveniently participate from various locations.

LCME accreditation visits scheduled for March and April 2020 were at first delayed, which left medical schools in the queue for a visit in limbo. Then, in May 2020 the LCME posted on their website that they had shifted the process to a virtual format and rescheduled these schools’ visit to June, July, and August 2020. Conducting accreditation visits virtually was a novel experience, and with this, new needs emerged for medical schools. Electronic sharing of documents had already been a requirement, so this did not present an issue. And, since an in-person visit of medical school facilities could not be accomplished, this task was replaced with a virtual tour, which was already available at our school via the internet to showcase our campus and medical student experiences. Yet, many other unanticipated difficulties surfaced during preparation for the virtual visit.

After the LCME confirmed that our accreditation visit would be moved to an online arena, we felt it was critical to test our technology capacities. A virtual visit meant moving nearly 100 participants into back-to-back online meetings over the course of a few days to succinctly answer questions posed by the survey team to corroborate evidence. The agenda is very tight and there is no room for technology issues as one may encounter in an ordinary web-based meeting. At our medical school, the FAL was responsible for preparation of the accreditation visit with support from the SVC and a task force. Therefore, the FAL began holding countless online meetings to practice with each group who would meet with the survey visit team. Position in front of the camera, projection of voice, and focused responses were points of emphasis. Each group identified the order in which they would conduct introductions. We discussed sharing the “floor” so all administrators, faculty, residents, and students (participants) had an opportunity to speak with the team. Then, we decided to mimic a virtual visit, not once but twice. Our medical school conducted its first mock survey visit with a team of external reviewers seven months before the accreditation visit where participants were able to practice a pseudo-high stakes meeting in the online environment using the same platform we planned to use during the LCME visit. The FAL and SVC used information gleaned from this first mock visit to determine what worked well and what adjustments were needed.

The LCME did not require a particular platform for the virtual visit, so we were able to use the video conferencing system employed by our institution. During the mock visit, participants were asked to sign in to their online meeting 15–30 minutes in advance to ensure their connection was reliable and they were in the virtual waiting room prepared to meet the external team of reviewers. Participants practiced introductions and took turns answering sample questions. Over the course of the two days of sessions during this initial mock visit, we quickly realized that personal devices made it difficult for IT to troubleshoot problems. Several participants were not on the university server and had bandwidth issues. Some devices were outdated and slow, which negatively impacted their connection. Other challenges included poor audio and visual quality that made it difficult for the participant to interact with the external team.

Aside from hardware and software issues, our medical school experienced additional problems during the mock visit. While participants were told to proceed in sessions as if this were the actual visit, some demonstrated overly casual behavior, wore very casual attire, or were disrupted by children or colleagues in the background. It appeared that some participants had their email account open, which was distracting and diverted their attention from the conversation during the meeting. Also, a few attempted to login to the session after it had already begun. The mock team secretary did not admit these participants into the virtual meeting to replicate what may happen in the actual visit, as this individual controls who is permitted to enter each session.

To solve the issues we encountered following the first mock visit, we moved all meetings for the accreditation visit to a physical location with appropriate social distancing, face masking, and sanitizing between sessions for safety. The medical school provided laptops in two large lecture rooms and staggered meetings between the two spaces to sanitize the computers and surfaces before use by the next group. We had extra laptops on-hand and powered on in the event that a few malfunctioned. The physical location most effectively allowed IT personnel to support participants because they were in-person and using reliable university devices. Two IT staff floated between the meeting rooms to immediately assist if there were any issues prior to the start of each meeting.

One month before the LCME accreditation survey visit, we conducted a second mock visit to fine tune procedures and ensure that participants felt prepared. We found that all the logistical changes we made were effective in eliminating the problems we experienced with devices, connectivity, audio, and distractions during the first mock visit. A one-page sheet of helpful tips was projected in the rooms to remind participants of which session they were in, their order of introductions, to mute when not speaking, how to login, and what to do if they could not connect. IT and room monitors were always available in the room prior to a session, and outside the doors once the meetings began.

Given our experience and lessons learned, below are recommendations to consider when planning for a virtual visit. These components may seem trivial but were necessary to address for a smooth virtual visit.

## Tips

1. Discuss the agenda and expectations with the team secretary, and exchange cell phone numbers.

The FAL, SVC, and team secretary should have each other’s contact information to text or call should an issue arise that needs to be resolved quickly during the visit.

2. Hold a mock virtual visit.

The mock visit was an extremely beneficial test of the logistics of a virtual accreditation visit that exposed holes in our planning. We had many unforeseen troubles that had the potential to compromise the success of our visit.

3. Utilize a central location for the visit if possible, and ask participants to display their full name and title when logging in.

If a centralized location is not feasible (e.g., schools with regional campuses), participants should utilize a neutral background in a location free from distractions.

4. Instruct participants to check in with the school’s registration team 30 minutes prior to the session.

It is important that each participant can sign into the virtual waiting room in advance of the start time of their meeting with the team in case there are any login issues. We had a team of 10 staff onsite helping with logistics throughout the visit.

5. Minimize disruptions.

We required participants to leave personal laptops, devices, and belongings at registration to avoid disruptions during the meetings. Notifications should be turned off and apps closed so focus is solely on the meeting with the survey team.

6. Utilize headsets.

Personal headsets were given to each participant to keep in order to optimize audio quality during the meetings.

7. Provide the survey team with a practice session prior to the first meeting.

We suggest offering the LCME team a practice meeting the day before the actual visit so they can test the platform, make certain they have the appropriate software downloaded, and can connect from an address and server external to the institution.

## Conclusions

The virtual mock visits were invaluable based on our experience. We encourage investing in at least one mock visit to prepare participants and test the technology that will be used during the online accreditation visit. While we conducted two mock visits, much can be learned from just one test trial. We highly recommend conducting the virtual visit in a centralized location with institutional laptops connected to the school’s server for best results.

Implications for virtual survey visits in the post-pandemic future should be explored. This article only represents the experience of one medical school; yet we found the virtual experience to be very positive and significantly reduced the cost associated with survey visits. Online visits may present a new opportunity to those individuals who are interested in serving as an LCME team member but face travel limitations. In contrast, virtual visits may have shortcomings just like any other online meeting. For example, the virtual setting does not allow the team to be physically present on campus to experience the culture, and online conversations can often proceed more rigidly than when in-person where there are natural signals (e.g., body language). Further, online fatigue can pose a challenge for the team members and participants who are in multiple meetings.

There may be other pros and cons not identified in this article. In April 2022, the LCME announced that it will return to in-person for all full, provisional, and preliminary survey visits effective the 2022–23 academic year, whereas all limited visits will be conducted virtually. With some visits remaining virtual, and the potential for future disruptions that would necessitate reinstating an online process, it is important to investigate the perceptions of survey team members who conducted virtual survey visits for the LCME and learn how other institutions managed their virtual survey visit.

## Data Availability

No data are associated with this article.
