# Supplementary material for: Virtual medical education accreditation survey visits and viability for the future
Source: MedEdPublish (2016). 2023 Jan 20;12:39. Originally published 2022 May 27. [Version 2] doi: 10.12688/mep.19160.2 (PMC10090858; doi:10.12688/mep.19160.2)
Supplement: Peer review history

# Peer review: Reviewer response for version 2

**DOI:** 10.21956/mep.20926.r32910

**Published:** 2023-04-11

**Authors:** Monica M. Cuddy

**Affiliations:** 1 National Board of Medical Examiners, Philadelphia, PA, USA

The authors have mostly addressed my comments. I still wonder, though, about whether some of the tips are unique to virtual site meetings or just apply to virtual meetings in general (e.g., minimize disruptions, utilize headsets). The need for a virtual tour sets virtual site visits apart from virtual meetings, so that would be an interesting topic to explore in future work.

If evidence from practice is presented, are all the underlying source data available to ensure full reproducibility?

Partly

Is the topic of the practical tips discussed accurately in the context of the current literature

Yes

Are all factual statements correct and adequately supported by citations?

Yes

Are the conclusions drawn balanced and justified on the basis of the presented arguments?

Yes

Are arguments sufficiently supported by evidence from the published literature and/or the authors’ practice?

Partly

Reviewer Expertise:

Medical regulation, licensure and certification in the professions, assessment/measurement

I confirm that I have read this submission and believe that I have an appropriate level of expertise to confirm that it is of an acceptable scientific standard.

# Peer review: Reviewer response for version 2

**DOI:** 10.21956/mep.20926.r32909

**Published:** 2023-01-27

**Authors:** Michael A. Whitt, Aditi Kesari

**Affiliations:** 1 Department of Microbiology, Immunology and Biochemistry, University of Tennessee Health Science Center, Memphis, TN, USA; 2 Department of Medical Education, University of Tennessee Health Science Center, Memphis, TN, USA

The authors have addressed our concerns and have made the appropriate revisions.

If evidence from practice is presented, are all the underlying source data available to ensure full reproducibility?

Yes

Is the topic of the practical tips discussed accurately in the context of the current literature

Yes

Are all factual statements correct and adequately supported by citations?

Partly

Are the conclusions drawn balanced and justified on the basis of the presented arguments?

Yes

Are arguments sufficiently supported by evidence from the published literature and/or the authors’ practice?

Partly

Reviewer Expertise:

Virology, Medical Education

We confirm that we have read this submission and believe that we have an appropriate level of expertise to confirm that it is of an acceptable scientific standard.

# Peer review: Reviewer response for version 1

**DOI:** 10.21956/mep.20523.r32408

**Published:** 2022-08-19

**Authors:** Monica M. Cuddy

**Affiliations:** 1 National Board of Medical Examiners, Philadelphia, PA, USA

This manuscript (classified as a “Practical Tips” contribution) focuses on one medical school’s experiences with preparing for and undergoing a virtual LCME accreditation site visit. The topic is timely given the pandemic and likely will be of interest to the MedEdPublish readership.

 The authors claim that even though LCME accreditation site visits are no longer virtual, knowing how best to handle a virtual accreditation site visit is useful for potential future instances where virtual site visits are again required. This claim is sound and provides a reasonable rationale for the current manuscript. My main concern with the manuscript has to do with the unique features of an LCME accreditation visit. More information and discussion about what constitutes a visit, and how a virtual visit is a unique form of a virtual meeting seems warranted. Without it, the tips are just tips for a successful virtual meeting, which is less interesting and potentially less helpful than tips specifically for a virtual accreditation site visit. That said, it could be useful to include a few tips that could be gleaned from the documented experiences, but were not implemented. Also, some information about who made the decisions about how to approach the virtual site visit would be good to add, as well as the processes for making those decisions.

If evidence from practice is presented, are all the underlying source data available to ensure full reproducibility?

Partly

Is the topic of the practical tips discussed accurately in the context of the current literature

Yes

Are all factual statements correct and adequately supported by citations?

Yes

Are the conclusions drawn balanced and justified on the basis of the presented arguments?

Yes

Are arguments sufficiently supported by evidence from the published literature and/or the authors’ practice?

Partly

Reviewer Expertise:

Medical regulation, licensure and certification in the professions, assessment/measurement

I confirm that I have read this submission and believe that I have an appropriate level of expertise to confirm that it is of an acceptable scientific standard, however I have significant reservations, as outlined above.

# Response

**Published:** 2023-01-10

**Authors:** Lori DeShetler

**Affiliations:** The University of Toledo, USA

Thank you for your review. As the author of this article, I have made the following revisions per your feedback:

- A description was added about how a virtual visit is different than a typical online meeting in paragraph nine.
- The author clarified who made decisions about how to approach the virtual visit and the process for making those decisions.
- Seven tips were added that are specific to a virtual accreditation survey visit.

# Peer review: Reviewer response for version 1

**DOI:** 10.21956/mep.20523.r32124

**Published:** 2022-07-07

**Authors:** Michael A. Whitt, Aditi Kesari

**Affiliations:** 1 Department of Microbiology, Immunology and Biochemistry, University of Tennessee Health Science Center, Memphis, TN, USA; 2 Department of Medical Education, University of Tennessee Health Science Center, Memphis, TN, USA

The manuscript by DeShetler entitled “Virtual medical education accreditation survey visits and viability for the future” covers an important and timely topic on virtual LCME site visits through the experience of a virtual survey visit at the University of Toledo. There is limited literature on virtual LCME site visits during the COVID-19 pandemic and the information provided in this manuscript provides useful information that can guide different medical schools going through this process.  Even though LCME has gone back to in-person visits, the limited survey visits still remain virtual. Also, having a plan of action for future unforeseeable circumstances is always beneficial. Thus, this topic holds merit. The author provides a good background while citing some virtual accreditation-visit experiences at other nursing and pharmacy schools. The author also shares the benefits and challenges related to virtual LCME meetings. The author proposes to share some tips that could be beneficial to other schools undergoing this process.

 While some tips shared by the author could be useful to some schools, the author could provide some additional insight, so that the tips can be more comprehensive and widely applicable. The way the tips section currently reads makes it appear more like a continuation of the previous section where the experience at their school is shared. The author does acknowledge that the article is based on the experience of one school in the abstract. Hence, discussing the pros and cons of the suggested tips would be beneficial.

 For example, during the mock visit experience at their institution, they faced some technical and behavioral issues. The participants were instructed to be present at a single physical location for the actual site visit. They were provided with laptops and headsets and had on-site IT personnel to address issues that may arise during the visit. In this section, the author could provide more details on logistics and planning, such as the total number of participants involved in the survey visit and the number of team members overseeing these logistics (apart from the two IT staff). This may help the readers evaluate the feasibility of implementing these suggestions at their schools. The author could also suggest alternative solutions to address the issues they faced at their institution. In that context, the author could weigh in on how things could be different at schools that have regional campuses. Additionally, the author cites a study in the introduction, in which the preceptors in the field of pharmacy were found to be in favor of virtual accreditation visits. Can that conclusion be factored into this discussion?

 Additional suggestions include:

1. The tip by Phan and Radovich (cited in the introduction) about the discussion between the director and lead appraiser being the first step can be reiterated in the tips section.
2. The introduction can be further improved in terms of general organization. Removing some redundancies can help improve the flow of the manuscript. For example, the point about travel costs associated with the visits has been repeated four times in the manuscript. 
  For example:
  - In paragraph 4: “An obvious benefit to conducting the visits virtually was the reduction of travel costs and time.”
  - Paragraph 5: “A considerable amount of expenses was incurred by the survey team for airfare and lodging, and the medical school was responsible for funding the team’s ground transportation and meals (breakfast and lunch) during the visit.”
  - Paragraph 6: “Travel costs associated with accreditation visits were eliminated and physical accommodations were no longer needed.”
  - Concluding paragraph: “yet we found the virtual experience to be very positive and significantly reduced the cost associated with survey visits.”
3. Similarly, the point about rescheduling and shifting LCME site visits to virtual format in May 2020 is discussed twice in the manuscript. It can probably be discussed in detail in the introduction, and just alluded to in the latter paragraph, if required.
4. In many places, references are not provided.  For example, provide citations for:
   
  - “The LCME decided in May 2020 to convert upcoming survey visits to online”.
  - “Medical schools appoint a Faculty Accreditation Lead (FAL) and Survey Visit Coordinator (SVC) who plan the visit and organize committees to conduct a self-study and draft the survey package”.
  - “Despite the number of articles that surfaced from other disciplines, at the time of writing this article there are yet to be any studies about the impact of COVID-19 on medical education accreditation visits by the LCME.”
5. The author should point out that the LCME team secretary controlled who was let into the meeting with the survey team.  A reference is made in the last sentence before the Tips section that anyone who logged in late was not allowed into the meeting with the survey team, but we also had instances where the team secretary “forgot” to let someone in who was present in the waiting room.  Here, and throughout the meeting, the FAL communicated with the team secretary via text messages.  It would be helpful to point out that text messaging between the FAL and team secretary can be beneficial for schools needing to prepare for an upcoming visit.
6. Another suggestion for schools with regional campuses where it is not possible to have all group members in the same room is that we asked all participants to use a standard, non-distracting virtual background that included the individual’s name and title.  This allowed the survey visit team to see who was speaking and what their role was without having to re-introduce themselves during the meeting.
  - An additional issue that arose during this reviewers’ virtual survey visit was that the LCME team secretary adjusted the order of the meetings a few days before the virtual visit which resulted in some clinical faculty not being able to participate due to conflicts at the adjusted time despite having release time build into their schedule when the FAL sent out the “final” agenda.

If evidence from practice is presented, are all the underlying source data available to ensure full reproducibility?

Yes

Is the topic of the practical tips discussed accurately in the context of the current literature

Yes

Are all factual statements correct and adequately supported by citations?

Partly

Are the conclusions drawn balanced and justified on the basis of the presented arguments?

Yes

Are arguments sufficiently supported by evidence from the published literature and/or the authors’ practice?

Partly

Reviewer Expertise:

Virology, Medical Education

We confirm that we have read this submission and believe that we have an appropriate level of expertise to confirm that it is of an acceptable scientific standard, however we have significant reservations, as outlined above.

# Response

**Published:** 2023-01-10

**Authors:** Lori DeShetler

**Affiliations:** The University of Toledo, USA

Thank you for your review. As the author of this article, I have made the following revisions per your feedback:

- For better flow, the paragraphs detailing the author's experience were moved prior to the Tips section. Then, a list of seven suggestions were added to be more comprehensive and widely applicable to the audience.
- Details on the logistics and planning (i.e., total number of participants involved in the survey visit and number of team members overseeing these logistics) were added.
- Tip #3 accounts for schools with regional campuses.
- Tip #1 reiterates the tip by Phan and Radovich about the discussion between the director and lead appraiser (team secretary).
- Redundancies were removed regarding travel costs and the rescheduling and shifting LCME survey visits.
- References were provided for the three bullets suggested in reviewer comment #4.
- The author clarified that the team secretary controls who is let into the meetings.
- Tip #1 recommends that the director exchange cell phone numbers with the team secretary should an issue arise that needs to be resolved quickly during the visit.
- Tip #3 addresses reviewer suggestion #6 regarding display of non-distracting virtual backgrounds.
